# Mitogenomics of historical type specimens clarifies the taxonomy of Ethiopian *Ptychadena* Boulenger, 1917 (Anura, Ptychadenidae)

**DOI:** 10.3897/zookeys.1070.66598

**Published:** 2021-11-12

**Authors:** Jacobo Reyes-Velasco, Sandra Goutte*, Xenia Freilich, Stéphane Boissinot

**Affiliations:** 1 New York University Abu Dhabi, Saadiyat Island, Abu Dhabi, UAE New York University Abu Dhabi Abu Dhabi United Arab Emirates; 2 Department of Biology, Queens College, City University of New York, Flushing, NY, USA City University of New York Flushing United States of America

**Keywords:** Grass frogs, Historical DNA, *Ptychadena*, taxonomy, type specimens

## Abstract

The taxonomy of the *Ptychadenaneumanni* species complex, a radiation of grass frogs inhabiting the Ethiopian highlands, has puzzled scientists for decades because of the morphological resemblance among its members. Whilst molecular phylogenetic methods allowed the discovery of several species in recent years, assigning pre-existing and new names to clades was challenged by the unavailability of molecular data for century-old type specimens. We used Illumina short reads to sequence the mitochondrial DNA of type specimens in this group, as well as ddRAD-seq analyses to resolve taxonomic uncertainties surrounding the *P.neumanni* species complex. The phylogenetic reconstruction revealed recurrent confusion between *Ptychadenaerlangeri* (Ahl, 1924) and *P.neumanni* (Ahl, 1924) in the literature. The phylogeny also established that *P.largeni* Perret, 1994 represents a junior synonym of *P.erlangeri* (Ahl, 1924) and distinguished between two small species, *P.nana* Perret, 1994, restricted to the Arussi Plateau, and *P.robeensis* Goutte, Reyes-Velasco, Freilich, Kassie & Boissinot, 2021, which inhabits the Bale Mountains. The phylogenetic analyses of mitochondrial DNA from type specimens also corroborate the validity of seven recently described species within the group. Our study shows how modern molecular tools applied to historical type specimens can help resolve long-standing taxonomic issues in cryptic species complexes.

## Introduction

In the highlands of Ethiopia, frogs of the genus *Ptychadena* Boulenger, 1917 form a monophyletic radiation known as the *Ptychadenaneumanni* species complex (Freilich et al. 2014). The molecular evolution of this group has been studied extensively, establishing it as a model system to study lineage diversification, speciation and biogeography in the region (Mengistu 2012; Freilich et al. 2014, 2016; Smith et al. 2017a; Reyes-Velasco et al. 2018). As in other regions of Africa, species of the genus *Ptychadena* of the Ethiopian highlands are difficult to distinguish from one-another based on morphological features alone (Poynton 1970; Bwong et al. 2009; Dehling and Sinsch 2013). Five *Ptychadena* species from the Ethiopian highlands were originally described based on morphology: *P.neumanni* (Ahl, 1924), *P.erlangeri* (Ahl, 1924), *P.cooperi* (Parker, 1930), *P.nana* Perret, 1980 and *P.wadei* Largen, 2000. A sixth species, *P.largeni*, was described by Perret (1994), but later synonymized with *P.neumanni* by Largen (2001). While some of the original descriptions allow for unambiguous species identification (e.g., *P.cooperi* and *P.harenna* Largen, 1997), assigning names to most of the lineages is challenging because some of the original discriptions do not provide diagnostic characters since the type series contain specimens belonging to different species (see Goutte et al. 2021).

Several authors found substantial morphological variation among populations of *P.neumanni*, which led them to suggest that this taxon consisted of multiple species (Perret 1994; Largen 1997, 2001). Freilich et al. (2014) used mitochondrial and nuclear loci to study the evolutionary history of the group, and their results showed that *P.neumanni* in fact comprised five distinct taxa, which did not form a monophyletic group. The authors refrained from describing the potential new species they identified because they were not able to compare their specimens with the type specimens of previously described species. Instead of providing new names, they assigned numbers to each one of the undescribed taxa they identified with their genetic analyses (i.e., *Ptychadena* cf. *neumanni 1*–*5*). Smith et al. (2017a) re-analyzed the combined molecular datasets of Mengistu (2012) and Freilich et al. (2014), as well as sequences from 30 new specimens, and recovered the same five highland taxa Freilich et al. (2014) had identified. Smith and colleagues then assigned new or pre-existing taxonomic names to each one of those lineages. However, they did not compare their material to the type specimens of previously described species in their morphological or molecular analyses and it thus remained unclear to which genetic lineage the names *P.neumanni*, *P.erlangeri*, *P.largeni* and *P.nana* should be assigned (Reyes-Velasco et al. 2018).

Recently, Goutte et al. (2021) revised the taxonomy of the group, based on morphology, molecular data, and call analyses and described four new species corresponding to four genetic lineages identified by Freilich et al. (2014) and Reyes-Velasco et al. (2018): *Ptychadenabeka* Goutte, Reyes-Velasco, Freilich, Kassie & Boissinot, 2021, *P.delphina* Goutte, Reyes-Velasco, Freilich, Kassie & Boissinot, 2021, *P.doro* Goutte, Reyes-Velasco, Freilich, Kassie & Boissinot, 2021 and *P.robeensis* Goutte, Reyes-Velasco, Freilich, Kassie & Boissinot, 2021.

**Table 1.** Taxonomic history of the *Ptychadenaneumanni* species complex.

Species	Author, year	Largen, 2001	Freilich et al. 2014	Smith et al. 2017	Reyes-Velasco et al. 2018	Goutte et al 2021 & this study
*P.neumanni*	Ahl, 1924	*P.neumanni / P.erlangeri*	*P.erlangeri*	*P.erlangeri*	*P.erlangeri*	*P.neumanni*
*P.erlangeri*	Ahl, 1924	*P.neumanni / P.erlangeri*	*P.* cf. *neumanni 2*	*P.largeni*	*P.* cf. *neumanni 2*	*P.erlangeri*
*P.cooperi*	Parker, 1930	*P.cooperi*	*P.cooperi*	*P.cooperi*	*P.cooperi*	*P.cooperi*
*P.nana*	Perret, 1980	*P.nana*	-	-	*P.* cf. *Mt. Gugu*	*P.nana*
*P.largeni*	Perret, 1994	*P.neumanni*	*P.* cf. *neumanni 2*	*P.largeni*	*P.* cf. *neumanni 2*	*P.erlangeri*
*P.harenna*	Largen, 1997	*P.harenna*	*P.harenna*	*P.harenna*	*P.harenna*	*P.harenna*
*P.levenorum*	Smith et al. 2017b	*P.neumanni*	*P.* cf. *neumanni 3*	*P.levenorum*	*P.* cf. *neumanni 3*	*P.levenorum*
*P.goweri*	Smith et al. 2017b	*P.erlangeri*	*P.* cf. *neumanni 4*	*P.goweri*	*P.* cf. *neumanni 4*	*P.goweri*
*P.amharensis*	Smith et al. 2017b	*P.neumanni / P.erlangeri*	*P.* cf. *neumanni 5*	*P.amharensis*	*P.* cf. *neumanni 5*	*P.amharensis*
*P.beka*	Goutte et al. 2021	*P.neumanni / P.erlangeri*	*P.* cf. *neumanni 1*	*P.neumanni*	*P.* cf. *neumanni 1*	*P.beka*
*P.delphina*	Goutte et al. 2021	-	*P.erlangeri*	*P.erlangeri*	*P.* cf. *erlangeri Metu*	*P.delphina*
*P.doro*	Goutte et al. 2021	-	*P.erlangeri*	*P.erlangeri*	*P.* cf. *erlangeri Gecha*	*P.doro*
*P.robeensis*	Goutte et al. 2021	-	*P.nana*	*P.nana*	*P.nana*	*P.robeensis*

**Figure 1.**
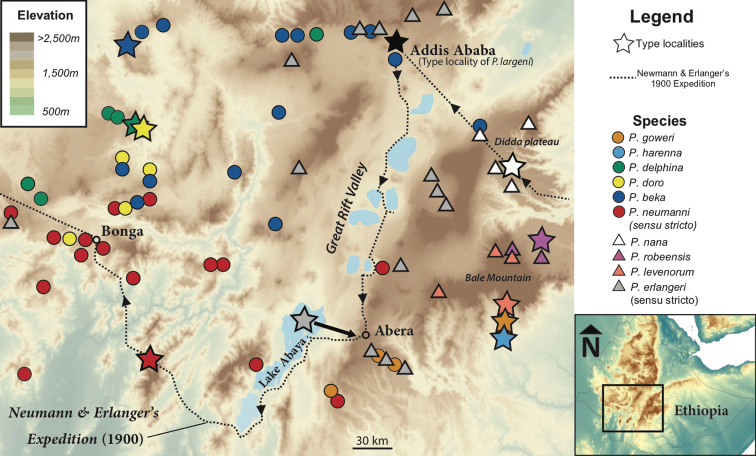
Map of Ethiopia showing localities of individuals in the *Ptychadenaneumanni* species complex used in this study. Samples with genetic data are represented by different colored circles (*P.neumanni* species group) or triangles (*P.erlangeri* species group). Stars depict the approximate type localities of *P.neumanni* (red), *P.erlangeri* (grey), and *P.nana* (white). A black star represents Addis Ababa, the type locality of *P.largeni*, a junior synonym of *P.erlangeri.* The approximate route of Oscar Neumann and Carlo von Erlanger’s 1900 expedition in Abyssinia, during which the type specimens of all the above species were collected (except for *P.largeni*) is represented by a dashed line. Black arrow indicates the likely correct type locality for *P.erlangeri* as suggested by the authors (see Discussion).

In order to resolve the taxonomy and systematics of the group, we extracted DNA from formalin or spirit-fixed type specimens of the species from which only morphological data was available (*P.erlangeri*, *P.largeni*, *P.nana* and *P.neumanni*) and reconstructed partial mitochondrial genomes. These sequences were included in a molecular phylogeny, along with mitochondrial DNA (mtDNA) from more recently collected material included in Reyes-Velasco et al. (2018) and in recent species descriptions (Fig. 1; Smith et al. 2017b; *P.amharensis* Smith, Noonan & Colston, 2017, *P.goweri* Smith, Noonan & Colston, 2017 and *P.levenorum* Smith, Noonan & Colston, 2017). We compared our mtDNA phylogeny to one obtained from thousands of genome-wide SNPs obtained from ddRAD-seq (Reyes-Velasco et al. 2018) to test for congruence between mitochondrial genetic lineages and species. These analyses allowed the assignment of existing names to genetic lineages and the validation of the three new species described by Smith et al (2017b). Finally, four previously identified lineages were established as new species, which we describe as *P.beka*, *P.delphina*, *P.doro* and *P.robeensis* elsewhere (Goutte et al. 2021).

## Material and methods

### DNA extraction and sequencing of type specimens

We aimed to extract mtDNA from type specimens for which molecular data was unavailable. The types of *Ptychadenacooperi* and *P.harenna* were not sequenced, as these two species are easily distinguishable morphologically and there is no ambiguity regarding their taxonomic status (Goutte et al. 2021). We obtained authorization from the Museum für Naturkunde Berlin (**ZMB**) and the Museum d’Histoire Naturelle, Genève (**MHNG**) to sample a small amount of muscle or liver tissue from the type specimens of *Ptychadenaneumanni* (lectotype, ZMB 26879; paralectotype, ZMB 57183), *P.erlangeri* (holotype, ZMB 26887), *P.largeni* (paratypes, MHNG 2513-15 & 2513-56) and *P.nana* (holotype, ZMB 26878). Tissue sampling did not result in any major visible damage to the vouchers, as most tissue was taken from pre-existing incisions. Dissecting tools were cleaned with a 10% bleach solution before and after tissue extraction.

Although we could not find information about the mode of preservation used at the time of collection, it is likely that the type specimens had been fixed in formalin or some form of spirit, which renders the extraction of DNA challenging and requires a different protocol than when using fresh tissue. We followed the protocol described by Shedlock et al. (1997). In short, approximately ~5 mg of tissue (muscle or liver) was placed in a 2 mL tube and washed in 1X GTE buffer for four days, changing the buffer daily. The tissue was then incubated for four additional days at 65 °C in a 2 mL tube with 500 μL cell lysis buffer, 100 μL proteinase K, and 20 μL 1 mM DTT (dithiothreitol). Proteinase K was added daily until all the tissue was completely digested. A standard potassium acetate (KOA) DNA precipitation protocol was then followed. A detailed protocol for the DNA extraction is provided in the Suppl. material 1. To reduce the possibility of contamination, DNA extraction was carried out using new reagents in a marine biology lab that does not work with amphibian samples, and multiple negative controls (using deionized water) were used along the process.

DNA concentration was measured using a high sensitivity kit in a Qubit fluorometer (Life Technologies) and DNA fragment size distribution and concentration was estimated using a Bioanalyzer 7500 high sensitivity DNA chip (Agilent, Santa Clara, CA, USA). A NEBNext FFPE DNA Repair Mix (New England Biolabs) was used to repair damaged bases prior to library preparation, which was carried out with a NEB library preparation kit. The shredding step was skipped because of the fragmented nature of historical DNA. All libraries were pooled and sequenced on an Illumina NextSeq 550 (75 bp paired-end) at the Genome Core Facility of New York University Abu Dhabi, UAE. The FASTx Toolkit (Gordon and Hannon 2010) was used to remove Illumina adaptors and low-quality reads (mean Phred score < 20). The final average read length was 63 bp after trimming (Suppl. material 2). The program *FastQC* (Andrews 2010) was then used to observe if base composition was biased towards the end of the raw reads, which is a common phenomenon resulting from deamination (Dabney et al. 2013) when sequencing older DNA, however this was not observed. Summary statistics describing the sequencing data are available in Suppl. material 2: Table S1. All sequences are deposited in GenBank (MW375737–MW375766; Suppl. material 2: Table S2).

### Assembly of mitochondrial genomes

Whole mitochondrial genomes of the type specimens were assembled from the Illumina reads using MITObim (Hahn et al. 2013). MITObim uses an iterative baiting method to generate mitochondrial contigs from short reads. First, a published sequence of the mitochondrial genome of *Ptychadenamascareniensis* (Duméril & Bibron, 1841) (GenBank reference number JX564890) was used as the reference mitogenome, with the default program settings, except for a *k*-mer length of 21. The analysis was then run again using the resulting contigs from the first MITObim run. An additional 21 mitochondrial genomes from fresh samples collected by us of members of the *P.neumanni* species complex were also assembled following the same protocol (Suppl. material 2: Table S2). These samples were sequenced for another project on the genomics of the *Ptychadenaneumanni* species group (Hariyani et al. in prep.) following the sampling, tissue handling and DNA extraction protocols of Reyes-Velasco et al. (2018).

### Phylogenetic analysis of mtDNA

We reconstructed phylogenetic relationships within the *Ptychadenaneumanni* species complex using three different datasets. First, all 13 mitochondrial protein-coding genes and the rRNA 12S and 16S genes were used. No stop codon was found in the protein-coding sequences. We excluded tRNAs because in some cases these were not complete or were difficult to align. Because we did not have the complete mitochondrial genome for all species, we used a subset of genes which was obtained for all species as a second dataset. This dataset included the 12S and 16S rRNA, as well as the protein-coding gene cytochrome *c* oxidase I (*cox1*). In the last dataset, we included only the rRNA 16S, in order to be able to include as many individuals as possible. Alignments at each gene were performed in MAFFT v. 7 (Katoh and Standley 2013), and other ambiguously aligned regions were removed using the online server G-Blocks (Castresana 2000) with the least stringent options selected. Geneious v. 9.1.6 (Biomatters Ltd., Auckland, NZ) was used to manually trim any remaining poorly aligned regions and to ensure that protein-coding genes were in the correct reading frame. Our final concatenated datasets consisted of 15,708 bp for the dataset that included all genes and 2522 bp for the reduced dataset (12S, 16S and *cox1*).

The best-fit model of nucleotide evolution for each gene was selected using the Bayesian Information Criterion (BIC) in PartitionFinder v. 1.1.1 (Lanfear et al. 2012; Suppl. material 2: Table S3). Data was partitioned by gene and by codon position in protein-coding genes. All genes were concatenated using the program Sequence Matrix (Vaidya et al. 2011) and performed Bayesian phylogenetic inference (BI) in MrBayes v. 3.2.2 (Ronquist et al. 2012) on the CIPRES Science Gateway server (Miller et al. 2010).

For both datasets, the BI analyses consisted of four runs of 10^7^ generations, sampling every 1000^th^ generation, with four chains (three heated and one cold). Convergence between the runs was assessed by visually inspecting overlap in likelihood and parameter estimates between the runs, as well as effective sample sizes and potential scale reduction factor (PSRF) value estimates for each run using Tracer v. 1.6 (Rambaut et al. 2014). Individual runs converged by 10^5^ generations, based on the PSRF, so the first 25% of each run were discarded as burn-in. The runs were combined, and the resulting tree was visualized in FigTree v. 1.4.2 (Rambaut 2014).

### Analyses of ddRAD-seq data

In a previous study (Reyes-Velasco et al. 2018), we sequenced thousands of loci from across the genome of all known lineages of *Ptychadena* inhabiting the Ethiopian highlands (12 putative species, 105 individuals) using double digest restriction-site associated DNA sequencing (ddRAD-seq). Here we briefly describe the methods used as more details were provided in our original article.

Individuals of the *Ptychadenaneumanni* species complex were collected in the highlands of Ethiopia between 2011 and 2018. Tissue samples were extracted and stored in RNAlater or 95% ethanol. Genomic DNA was extracted with one of the following methods: with the use of a DNeasy blood and tissue kit (Qiagen, Valencia, CA), with the use of Serapure beads (Rohland and Reich 2012), or by standard potassium acetate extraction. DNA concentration was measured with a Qubit fluorometer (Life Technologies) so that DNA sample concentrations could be standardized. Genomic DNA was then digested with the enzymes SbfI and MspI (Peterson et al. 2012). Barcoded samples were size-selected between 400 and 550 base pairs using a Pippin Prep (Sage Science, Beverly, MA, USA), and attached to unique Illumina indices (Peterson et al. 2012). Libraries were sequenced on an Illumina HiSeq2500 (100 bp paired-end reads) at the Genome Core Facility of New York University Abu Dhabi, United Arab Emirates.

Ipyrad 0.6.17 (Eaton and Overcast 2020) was used to assemble loci *de novo* and create SNP datasets. After quality filtering, a total of ~158 million sequencing reads were retained, with a mean coverage of about 1.60 million reads, and a mean of ~11,400 RAD-tags per individual. We obtained between 800 and 2918 polymorphic loci and between 28,000–36,000 SNPs (Reyes-Velasco et al. 2018). The best model of evolution for our concatenated ddRAD-seq dataset was estimated with using BIC in PAUP v. 4.0.a151 (Swofford 1993), which showed the GTR + I + G model as the most supported. Maximum likelihood (ML) was implemented in RAxML v. 8 (Stamatakis 2014) to infer evolutionary relationships between populations and species in this group. RAxML was performed with rapid bootstrapping in the CIPRES portal (Miller et al. 2010).

## Results

### DNA extraction, sequencing and assembly of mtDNA genomes

DNA was successfully extracted from the type specimens of *Ptychadenaneumanni*, *P.erlangeri*, *P.largeni* and *P.nana*. After quality filtering, a total of ~1.1 billion reads were retained, with highly variable coverage across individuals (49–359 million reads; Suppl. material 2: Table S1). The complete mitochondrial genome was recovered for five out of the six type specimens and a partial sequence was obtained for the holotype of *P.nana*. The total number of reads from the type specimens that mapped to the reference mitochondrial genome ranged between 802 (*P.nana* holotype) to > 900,000 (*P.erlangeri* holotype; Suppl. material 2: Table S1). We found no correlation between the amount of DNA recovered and the final number of reads for each specimen.

### Phylogenetic analysis

**Figure 2.**
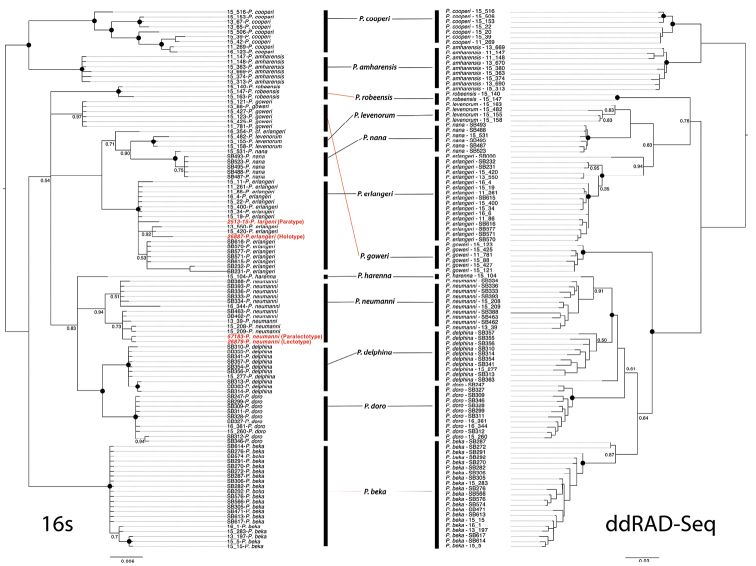
Comparisons of the topologies of the mitochondrial rRNA 16S (left) and ddRAD-seq (right) for members of the *Ptychadenaneumanni* species complex. Type specimens are indicated in red in the 16S phylogeny. Red lines indicate clades that differ in their placement between 16S and ddRAD-seq, however, the assignment of individuals to a particular species is identical between datasets. Numbers at nodes represent posterior support (pp), while black dots represent nodes with posterior support of 1.

**Figure 3.**
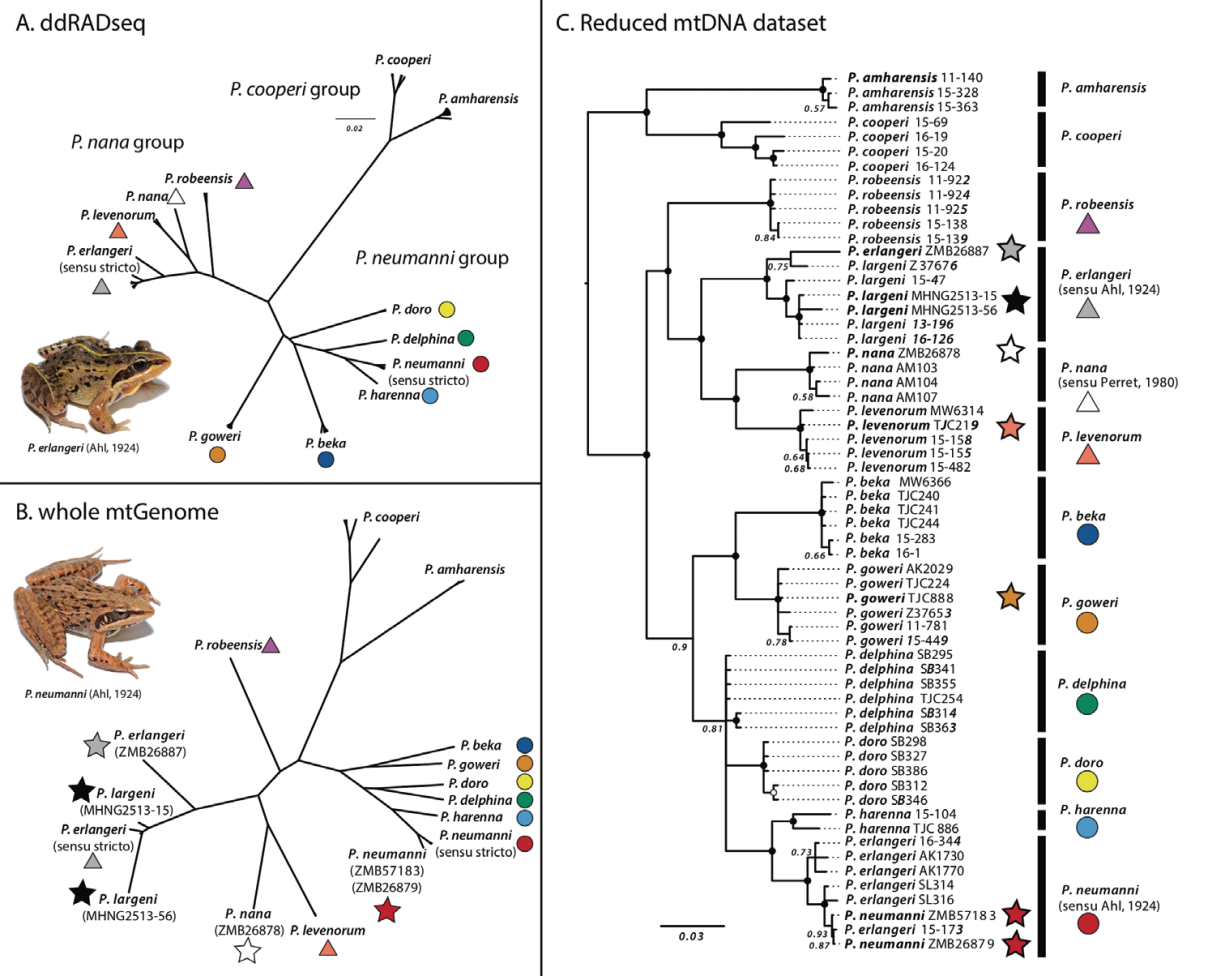
Phylogenetic inference of members of the *Ptychadenaneumanni* species complex based on mtDNA and ddRAD-seq data **A** unrooted UPGMA tree of members of the *P.neumanni* species complex based on 2182 SNPs obtained with ddRAD-sequencing **B** unrooted Bayesian phylogenetic inference based on the complete mitochondrial genomes of members of the group **C** bayesian phylogenetic inference based on the concatenated sequences of the 12S and 16S rRNA and *cox1*. Black circles represent nodes with a posterior support of 1. Names in bold indicate type specimens, while stars indicate historical type specimens sequenced here and are color-coded as in Figure 1. Photographs represent members of the *P.neumanni* species complex; *P.erlangeri* (top), *P.neumanni* (bottom).

As the assignment of species names to genetic lineages was based on mitochondrial sequences, we first verified that the assignment of individuals to genetic lineages using mitochondrial markers was congruent with that obtained using genome-wide loci from ddRAD-seq (Fig. 2). The clustering of 105 individuals based on nuclear SNPs and on mitochondrial sequences was perfectly congruent (Figs 2, 3a, b); thus, we considered that, at least in this particular case, mitochondrial DNA was sufficient to assign samples to the genetic clusters identified by previous authors (Freilich et al. 2014; Smith et al. 2017a; Reyes-Velasco et al. 2018; Goutte et al. 2021).

Our phylogenetic analysis based on three mitochondrial genes (Fig. 3c) recovered 11 mitochondrial clusters corresponding to 11 of the 12 genetic lineages, with high support. The only exception was *P.delphina*, for which mitochondrial sequences did not form a clade (Fig. 3c). The lectotype and paralectotype of *P.neumanni* (ZMB 26879 and ZMB 57183) were nested with strong support (PP = 1) within the clade that had been referred to as *P.erlangeri* by multiple authors (Freilich et al. 2014; Smith et al. 2017a; Reyes-Velasco et al. 2018). The holotype of *P.erlangeri* (ZMB26887) and the paratypes of *P.largeni* (MHNG 2513–15 and 2513–56) all grouped with strong support (PP = 1) with individuals previously assigned to either P.cf.neumanni 2 (Freilich et al. 2014) or *P.largeni* (Smith et al. 2017a). This result demonstrates that *P.largeni* represents a junior synonym of *P.erlangeri*.

The holotype of *Ptychadenanana* (ZMB26878) did not group with individuals from the Bale Mountains identified as *P.nana* by previous authors (Freilich et al. 2014; Smith et al. 2017a; Reyes-Velasco et al. 2018; Fig. 3c) but instead grouped with individuals from the Arussi Plateau (= Didda Plateau; Fig. 1), which corresponds to the type locality for the species (Perret 1980). The holotypes for the three species described by Smith et al. (2017a), *P.amharensis*, *P.goweri* and *P.levenorum*, clustered with genetic lineages that did not include sequences derived from historical type specimens (Figs 2, 3) and thus constitute valid species. Finally, four lineages did not include any type specimen of species described by Smith et al. (2017a) or prior and the corresponding species were recently described by Goutte et al. (2021; *P.beka*, *P.delphina*, *P.doro* and *P.robeensis*). Notably, the lineage corresponding to *P. cf. neumanni 1* of Freilich et al. (2014), which was previously suggested to be conspecific with *P.neumanni* by Smith et al. (2017a), was in fact genetically distinct and corresponded to the recently described *P.beka*.

## Discussion

In this study, we used historical DNA from century-old type specimens to resolve the convoluted taxonomy of the *Ptychadenaneumanni* species complex. Our results established the correspondence between genetic lineages and species originally described on morphological characters only. This allowed us to correct recurrent taxonomic errors made by multiple authors since the descriptions of the first species of the group, and to define which lineages correspond to new species. In addition, we were able to confirm the validity of some recently described taxa (*P.goweri*, *P.amaharensis*, *P.levenorum*, *P.robeensis*, *P.delphina*, *P.doro* and *P.beka*) and to synonymize others (*P.largeni*). Perret (1994) described *Ptychadenalargeni* from specimens of *P.erlangeri* collected by Malcom J. Largen in Addis Ababa. Perret based his diagnosis of *Ptychadenalargeni* on the absence of continuous dorsal folds in males and a smaller body size than *P.erlangeri* or *P.neumanni*. However, Largen (1997) casted doubt on the validity of this species and eventually synonymized it with *P.neumanni* (Largen, 2001), even though he had originally assigned those individuals to *P.erlangeri*. Our results confirm that Largen’s original identification of the specimens he collected in Addis Ababa was correct and that *P.largeni* is a junior synonym of *P.erlangeri* and not of *P.neumanni*. Confusion in the taxonomy of the *Ptychadenaneumanni* complex arose from the difficulty to identify morphologically similar species and the absence of comparison between sequenced and type specimens. To assign species names to populations, multiple authors have relied on geographic localities (Freilich et al. 2014; Smith et al. 2017a; Reyes-Velasco et al. 2018). In many cases, however, type locality data may be insufficient to attribute species names, either because species distribution ranges overlap or because type locality information is unreliable. For example, the holotype of *P.erlangeri* was collected during Oscar Neumann and Carlo von Erlanger’s expedition to Abyssinia in 1900; with the type locality indicated as “Lake Abaya” (Ahl 1924). The lake is located at 1200 m a.s.l., which is substantially lower than any other known locality for members of the *P.neumanni* species complex (>1500 m a.s.l.; Largen and Spawls 2010) and seems an unlikely locality for a population of *P.erlangeri*. However, on their way to Lake Abaya, the expedition party spent some time in the village of Abera, which is located at ~2700 m a.s.l. (Neumann 1902). We believe that the holotype of *P.erlangeri* was either collected between Abera and Lake Abaya or at Abera itself, nearby which we have collected *P.erlangeri* (15 km SE; Fig. 1). Confusion emerging from imprecise type localities is inevitable for many specimens collected in such expeditions, which were the main source of scientific collections in past centuries. Systematists should thus take these inconsistencies into account and refer to the physical name-bearing types as the main source of information, rather than type locality data alone. The recent development of methods to sequence DNA from formalin-fixed historical specimens provides a unique opportunity to expand the use of type specimens, and to include them in molecular phylogenetic analyses (Ruane and Austin 2017). In recent years, multiple techniques have been developed to obtain mtDNA from historical museum specimens of amphibians, which has been fundamental in resolving long and convoluted taxonomic questions. These newly developed techniques include target enrichment of mitochondrial DNA (Rancilhac et al. 2020; Scherz et al. 2020) or the use of single-stranded libraries (Lyra et al. 2020; Straube et al. 2021). In the present study, developing capture probes or single-stranded DNA libraries was not needed to obtain enough DNA for sequencing. However, multiple factors might influence DNA preservation (Straube et al. 2021), and additional pre-sequencing preparation steps may be necessary for other historical specimens. The sequencing of museum material may not always be possible due to DNA damage, because extracting tissues would damage type specimens, the type specimens have been lost, or simply because these methods might be too costly for researchers. Yet, recent technical progress as well as decreased sequencing costs open new opportunities for the use of museum specimens, thus highlighting the importance of museum collections in modern taxonomic research.

## Supplementary materials

Supplementary material 1 Detailed guidelines for the DNA extraction from museum specimens used in this study Data type guidelines https://binary.pensoft.net/file/610382This dataset is made available under the Open Database License (http://opendatacommons.org/licenses/odbl/1.0/). The Open Database License (ODbL) is a license agreement intended to allow users to freely share, modify, and use this Dataset while maintaining this same freedom for others, provided that the original source and author(s) are credited.Jacobo Reyes-Velasco, Sandra Goutte, Xenia Freilich, Stéphane Boissinot

Supplementary material 2 Table S1 Data type molecular data Explanation note Number of raw reads that passed quality filtering, average read length and number of reads that mapped to the mitochondrial genome for the type specimens sequen. https://binary.pensoft.net/file/610383This dataset is made available under the Open Database License (http://opendatacommons.org/licenses/odbl/1.0/). The Open Database License (ODbL) is a license agreement intended to allow users to freely share, modify, and use this Dataset while maintaining this same freedom for others, provided that the original source and author(s) are credited.Jacobo Reyes-Velasco, Sandra Goutte, Xenia Freilich, Stéphane Boissinot
