# Supplementary material for: Mitogenomics of historical type specimens clarifies the taxonomy of Ethiopian Ptychadena Boulenger, 1917 (Anura, Ptychadenidae)
Source: Zookeys. 2021 Nov 12;1070:135–49. doi: 10.3897/zookeys.1070.66598 (PMC8604866; doi:10.3897/zookeys.1070.66598)
Supplement: Supplementary material 2 — Table S1 [file zookeys-1070-135-s002.docx]

**Supplementary Methods**

**Protocol for extraction of DNA from Museum specimens**

Prepared by Jacobo Reyes-Velasco, modified from Shedlock et al. (1997)

1)  Take a small piece of tissue from the specimen, preferably liver or muscle that has not been in contact with the exterior. Cut it as small as possible.

2)  Place tissue sample in a 2ml Eppendorf tube and add **1.5mL of GTE buffer** (Thermo Fisher #J62597).

3)  Place tube in rotary shaker for 24 hours. Make sure that it is not shaking too fast.

4)  Change the GTE buffer and repeat step for another 24 hours.

5)  Change the GTE buffer once more and leave in shake for another 24 hours.

*(In total, the tissues should be in GTE buffer for approximately 2 to 3 days).*

6)  Remove all GTE buffer and allow sample to air dry completely (3-5 hours).

**Note: Avoid vortex mixing and other disturbances that can shred the DNA.**

*7)*Digest tissues in 500ul of **extraction buffer** at **65° for at least 24 hours**.

>Extraction buffer consist (for each sample):

1. 1M Tris-HCL - 50 μl
2. 0.5M EDTA – 100 μl
3. 10%SDS–50μl
4. 5MNaCl-10
5. DEPC H2O – 290 μl
6. 1M DTT (dithiothreitol) – 2 μl
7. Proteinase K (10mg/ml) – 100 μl

8)  Add additional 50ul of Proteinase K and 10ul of DNAse-free H20 after 10 hours.

9)  Digest tissue for up to four days, and keep adding proteinase K as needed.

10)Once the tissue is completely digested, add **187.5 μL of 5M KOAc** (potassium acetate) to each tube, vortex, and put in -80°C overnight (or longer).

11)Spin samples in centrifuge, max speed, room temperature, 10 minutes.

12)Transfer 900 ul of supernatant to a new 1.5mL tube using 1mL pipette. Do this relatively quickly so sediment doesn’t re-suspend. If this happens, spin samples again.

13) Add 600 μl of cold 100% isopropanol and 3 μl of **PureGene glycogen solution** (Gentra Systems, Inc.) to each tube to precipitate DNA. Mix gently, and leave for 5 minutes at room temperature.

14) Spin samples in centrifuge, max speed, room temperature, for 15 minutes.

15)Discard supernatant by decanting or with pipette (do quickly and re-spin samples if pellet does not stay in tube).

16) Add 150 μL of 80% ethanol to each tube, mix gently.

17) Spin samples in centrifuge, max speed, room temperature, 5 minutes.

18) Discard supernatant using 200 μL pipette (do quickly and re-spin if pellet does not stay in tube).

19) Air-dry DNA pellet (~15-30 minutes).

20) Add 30 μL of **PRE-HEATED** (56°C) 10 mM Tris-HCL. Leave to resuspend overnight before use.

21) Quantify DNA amount with Qubit High Sensitivity kit and DNA size in a Bioanalyzer High Sensitivity chip.

22) Store in -80°C until library preparation.

**References:**

Shedlock AM, Haygood MG, Pietsch TW, Bentzen P (1997) Enhanced DNA extraction and PCR amplification of mitochondrial genes from formalin-fixed museum specimens. Biotechniques 22: 394-400
